# Using Machine Learning to Evaluate Coal Geochemical Data with Respect to Dynamic Failures

**DOI:** 10.3390/min13060808

**Published:** 2023-06-09

**Authors:** David R. Hanson, Heather E. Lawson

**Affiliations:** CDC NIOSH Spokane Mining Research Division, Spokane, WA 99207, USA

**Keywords:** machine learning, coal, dynamic failure, burst, bump

## Abstract

Dynamic failure events have occurred in the underground coal mining industry since its inception. Recent NIOSH research has identified geochemical markers that correlate with in situ reportable dynamic event occurrence, although the causes behind this correlative relationship remain unclear. In this study, NIOSH researchers conducted machine learning analysis to examine whether a model could be constructed to assess the probability of dynamic failure occurrence based on geochemical and petrographic data. Linear regression, random forest, dimensionality reduction, and cluster analyses were applied to a catalog of dynamic failure and control data from the Pennsylvania Coal Sample Databank, cross-referenced with accident data from the Mine Safety and Health Administration (MSHA). Analyses determined that 7 of the 18 geochemical parameters that were examined had the biggest impact on model performance. Classifications based on logistic regression and random forest models attained precision values of 85.7% and 96.7%, respectively. Dimensionality reduction was used to explore patterns and groupings in the data and to search for relationships between compositional parameters. Cluster analyses were performed to determine if an algorithm could find clusters with given class memberships and to what extent misclassifications of dynamic failure status occurred. Cluster analysis using a hierarchal clustering algorithm after dimensionality reduction resulted in four clusters, with one relatively distinct dynamic failure cluster, and three clusters mostly consisting of control group members but with a small number of dynamic failure members.

## 1. Introduction

Prior works [1-4] have shown that coal seams that have experienced dynamic failures tend to have a correlation between failure occurrence and particular geochemical markers. The question of whether these markers are indicative of changes in (or effects on) the physical properties of the coal or rather are proxies for external features, such as depositional history and surrounding lithology, remains largely unanswered. Correlation does not necessarily imply causation, but observed empirical geochemical relations may serve as a practical technique to assess dynamic failure risks and to evaluate other confounders (such as coal rank, overburden thickness, regional stress orientation and magnitude, and mining design, among others). Researchers have developed numerous empirical relations in various attempts to predict other properties of interest [5], so it is reasonable to explore the possibility that dynamic failure propensity may be addressed in the same way.

With the explosive growth of ‘big data’, machine learning (ML) and artificial intelligence (AI) applications have been commensurately used to explore the correlations in these large data sets. These algorithms attempt to find patterns in the data, from which predictions or classifications may be made. However, as pointed out in McGaughey [6], the application of ML to geomechanics is not simple. Correlations can be especially complex. Thus, appropriate preconditioning and the careful selection of feature data are critical in order to determining which features correlate to a hazard, how they correlate to that hazard, and how assumptions on relationships between them can be confirmed or refuted. Additional research is thus required to ascertain why these correlations may exist.

ML applications in geohazard definition are now broadly utilized. Mira Geoscience offers an extensive software package, using both geomechanical features and their temporal variation, which can be applied in underground mines [7]. Other studies have compared the performance of several different ML algorithms to determine the best approach for forecasting rockbursts [8-11]. Several open-source software packages exist that allow researchers to select appropriate data and apply a model of their choice, including the package used in this study, known as Python Sklearn. However, there is no evidence that any of these methods have been specifically applied to examine the correlations between coal geochemical and compositional properties and rock mechanics problems.

The majority of ML works in the geohazard literature deal with features such as rock strength, Poisson’s ratio, microseismic event parameters, local geology, fault location, and rock quality designation (RQD). Models that use geochemical features are much less common. Toward this end, the analysis performed in this study uses geochemical data drawn from the Pennsylvania State Office of Coal (PSOC), cross-referenced with MSHA accident data after Lawson et al. [1] and supplemented with additional dynamic failure cases provided by Christopher Mark of the MSHA [12]. A binary categorical measure was applied to each coal record which indicated whether it came from a seam that had or had not experienced dynamic failure at any point in its history. It is important to note that this classification does not consider frequency, accident severity, mining methods, or any other attributes relevant to dynamic failure occurrence. Moreover, these data represent in situ accident occurrences, subject to many external and uncontrolled variables. These data consisted of 472 entries, each containing 37 descriptive features. These features included both numeric data and alphanumeric data and features that were computed from others in the set, and so were not independent. The objectives of these analyses were to:

1. Investigate whether machine learning techniques could be applied to a larger data set than that used in Berry et al. [2];
2. Apply other machine learning approaches to investigate cluster analysis and dimensionality reduction with regards to data structure and dynamic failure status;
3. Explore whether machine learning could provide a classifier of dynamic failure and control coals using those geochemical features that had the greatest impact on model outcomes.

## 2. Data and Methods

### 2.1. Description of the Data Set

Initially, Lawson Prime data consisted of 472 entries with 37 features, including 1 column for a data label (1 = dynamic failure and 0 = control) and 1 column for a PSOC sample number. Prior to performing machine learning analysis on these data, several pre-processing steps were performed. First, all alphanumeric data features were deleted, along with features that had null data. To avoid ‘multicollinearity’ or the use of interdependent variables, a correlation plot between features was examined. When a high correlation (or anti-correlation) exists, the features may not be independent. Hence, highly correlated features provide the same information, and so one should be dropped. Of the 37 initial features, 17 were then removed. These removals only address the most highly correlated variables, however, and many of the remaining variables retain some degree of interdependency. Based on the number of null data entries, 2 additional features were deleted, leaving 18. The final data set consisted of 468 points. Of these, 95 points were from coal seam samples that reported dynamic failure and the remaining 373 were considered the control group.

Coal rank is determined by differences in the calorific value, fixed carbon content, and volatile matter content, which in turn impact the physical properties of the coal. Lignite, for example, at one end of this spectrum, has a low calorific value and low fixed carbon, is relatively soft with a dull or earthy luster, and may exhibit poorly developed or absent cleating. Anthracite, by contrast, has high fixed carbon and a high calorific value, and is hard with an adamantine luster and very well developed cleating. The data span all commercially mined coal ranks. The majority, however, fall within the bituminous range with 215 or approximately 45% falling within the high-volatility bituminous A range.

The distribution of the final 468 data points, with respect to state location and rank, is given in Tables 1 and 2.

A quick look at these data show that the vast majority of cases were taken from the Appalachia Basin. In addition, most data points were from bituminous coals. This is true of both dynamic failure and control cases. Additionally, the discrepancy between the number of dynamic failure versus control cases renders the data available for analysis, imbalanced with the majority population falling within the control group.

Logistic regression and random forest analyses were performed on the scrubbed data set, assuming that the data were dichotomous (i.e., had or had not experienced dynamic failure), to see if an effective model indicating the potential of seam dynamic failures based on compositional features could be constructed. It must be emphasized that this analysis in no way seeks to replace the consideration of stresses, mining methods, overburden thickness, etc., as agents contributing to dynamic failures. Instead, by assuming that all other factors are equal, this analysis seeks to examine the possibility that compositional factors may provide an indication of whether some coals may have a higher propensity for dynamic failures.

The data set was then further examined for data structure using cluster analysis and dimensionality reduction. Cluster analysis was performed to see if an algorithmic application could extract the same clusters (or other clusters) as an analyst and to explore the data for relationships and structures not readily apparent in higher-dimensional space. Cluster analysis was performed using hierarchical density-based spatial clustering of applications with noise (HDBscan) [13]. Dimensionality reduction was performed using both principal component analysis (PCA) and t-distributed stochastic neighbor embedding (t-SNE), as described by Van der Maaten and Hinton [14].

### 2.2. Logistic Regression

After the number of features was reduced, the logistic regression package available in Python Sklearn [15] was applied. Numerous articles in the literature point out that in logistic regression, data normalization is not necessarily needed [16]. However, the different feature values retained for analysis in the Lawson Prime data set possessed values that varied by several orders of magnitude. This variation in data magnitude could potentially reduce or magnify their impact in both regression fitting and cluster analysis, and so normalization was applied. Tests were performed with no scaling or normalization, with data centering, data normalization, and data standardization. Centering provided the best results in terms of preliminary classification accuracy. This method was used to scale data in all subsequent work.

While models can be constructed using the entire feature set, this is not efficient and not all features may contribute to the logistic regression classification accuracy. It is important to try and select only the most significant features for regression based on defined criteria. Recursive feature elimination with cross-validation (RFECV) was applied to the edited Lawson Prime data set [15,17]. Recursive feature elimination involves a backward selection of predictors. First, a model is built using all the features and an importance score is computed for each. The least important is removed and the process is repeated. Based on this, a set of features that most effectively performs a classification is determined. These features can then be used to train a final model. The RFE algorithm used was stratified k-fold, with 2-fold cross-validation.

Another item to consider at this point is regularization. During RFECV, the best fits are determined by a maximum likelihood estimator. Regularization applies a penalty function to this expression, so the values of coefficients are driven smaller to prevent overfitting of the data [18,19].

Finally, prior to the application of RFECV and training of the classification model, the distribution of data must be considered. As data in the edited Lawson Prime set are asymmetric or imbalanced, the probability of rare dynamic failure events would most likely be underestimated [20-22]. To account for this asymmetry, a series of trials were conducted with RFECV where the majority class was down-sampled to create a balanced data set [23]. From the set of 373 control points, a randomly selected set of 86 was used in combination with 86 dynamic failure cases. A total of 3000 such cases were tested, and the highest scoring features were selected from these 3000 trials.

The 18 features applied in calculating the optimal number to be used for the Lawson data set are listed in Table 3.

**Figure 1.**
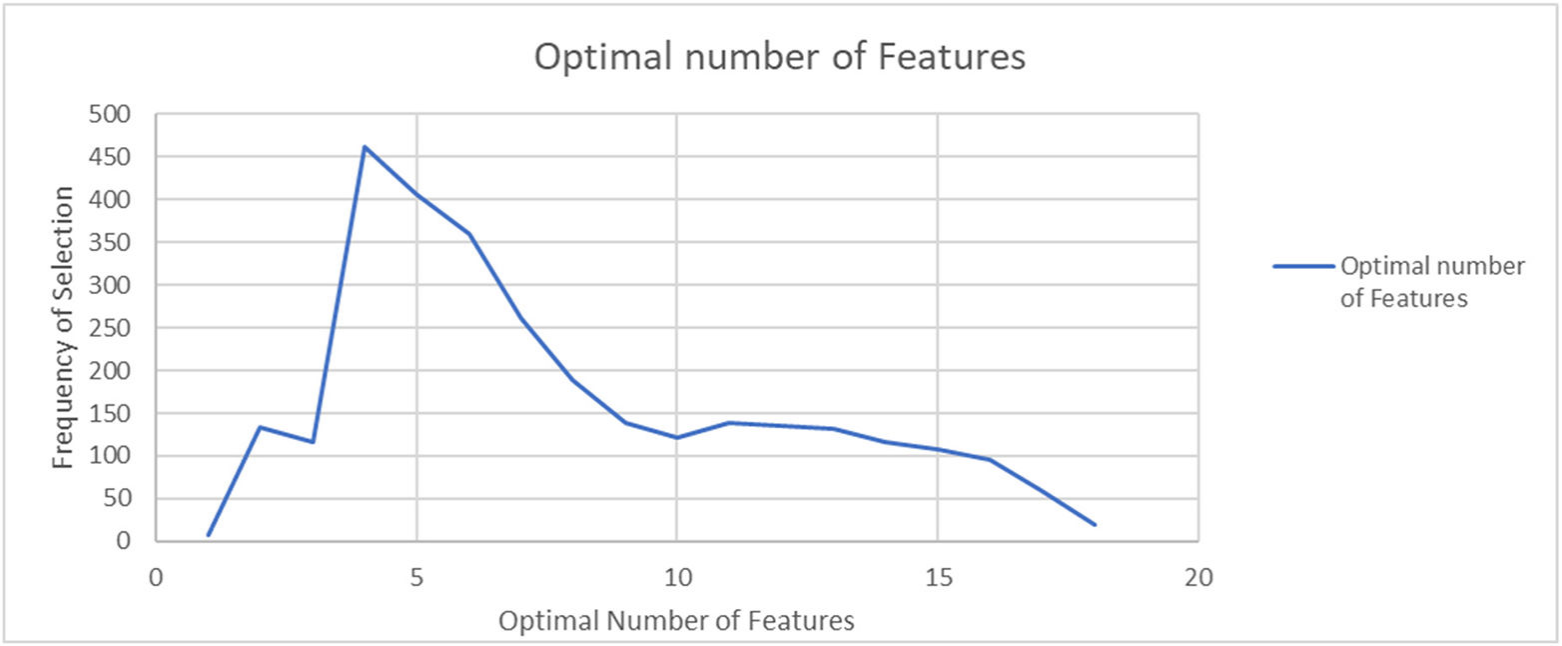
Optimal number of features selected from 3000 trials in the Lawson Prime data set. Four features were chosen most often in 462 out of 3000 trials.

Figure 1 shows how many times a particular number of features was chosen as optimal using RFECV. In the 3000 trials, 4 features were determined to be optimal in the highest percentage of trials (462 of the 3000 trials or 15.4% of the total). This was followed by 5 features in 406 trials, or 13.5% of the total. This plot shows how many features were selected as optimal, but not what those features were.

Examining the optimal features determined from RFECV, four features dominated the most common selection. Pyritic sulfur content was computed to have the highest ranking in 2997 of the 3000 trials. This was followed by organic sulfur content, oxygen content, and volatile matter content. These 4 features were selected to perform logistic regression. The data were split into training and testing sets, the model was trained, and this model was then applied to the test data set. Due to the large asymmetry between dynamic failure and control data entries, as with RFECV, the data were reduced to all dynamic failure entries and a similar number of randomly selected control points. Of the 180 points that were selected, 60% were chosen for training and 40% for testing. Again, a total of 3000 trials with a 60/40 train–test split were conducted.

The classifier was trained on 108 points (60%) and tested on 72 points (40%). A confusion matrix was constructed for each test, giving the number of true positives (TPs), true negatives (TNs), false positives (FPs), and false negatives (FNs). The confusion matrix averages from the 3000 trials are shown in Table 4. The number of true negatives should be 32 and the number of true positives should be 40. The average results show 32.8 true positives, 25.5 true negatives, 7.2 false negatives, and 6.5 false positives. Table 5 gives the precision, recall, and F-beta scores computed on each trial and averaged over the total number of trials for the Lawson Prime data. Over the 3000 trials, the average precision and recall were approximately 81%.

### 2.3. Random Forest Classification

There are several different approaches used to perform classifications in machine learning besides logistic regression, but random forest has several advantages over other learning algorithms. These include a smaller chance of overfitting as compared to decision trees, less training time, and high accuracy within large databases. Disadvantages include the possibility of overfitting on some data sets. The algorithm, as implemented in Python Sklearn, constructs multiple decision trees during training. The decision from the majority of these trees is then chosen as the final decision [24].

Several variants on a random forest application were tested, and the results were compared to each other and to the results from logistic regression. First, the random forest algorithm was applied to the Lawson Prime data using default program parameters. Second, since the data were imbalanced, decision threshold tuning [25,26] was applied to better address the correct classification of minority class data. Finally, to address the minority class and to reduce the chance of overfitting, a random hyperparameter search with cross-validation was applied [27]. While receiver operating characteristic (ROC) curves of true-positive rates versus false-positive rates can be produced, precision–recall curves are favored as a measure of algorithm effectiveness in the case of imbalanced data [28]. Because the data are imbalanced, the algorithm skill to predict the majority control cases (or a high number of true negatives) is of lower interest, and the cost of false negatives in dynamic failure classification is much higher than that of false positives. Thus, precision–recall curves, which provide more valuable information on classifier performance in the case of imbalanced data, were used here.

Recall and precision are given by:

(1) $$Recall=Sensitivity=\frac{TP}{TP+FN}$$

(2) $$Precision=\frac{TP}{TP+FP}$$

where TP=the number of true positives from the classifier; FN=the number of false negatives from the classifier; FP=the number of false positives from the classifier.

Thus, precision describes what proportion of positive identifications was actually correct. Recall quantifies what proportion of actual positives was correctly identified. Recall is then a measure which explains how well the model finds all of the minority class members [28].

The default decision threshold was set to 0.5, meaning that data points with normalized predicted class membership probabilities greater than or equal to 0.5 were assigned to Class 1 (dynamic failure positive), and those with a probability less than 0.5 were assigned to Class 0 (control cases). Tuning this decision threshold can help address the imbalance and produce better results in terms of classifications [26]. Results with both a default threshold and a tuned decision threshold were examined. When tuning the decision threshold, several metrics may be used to numerically evaluate the performance of the model: the G-mean, the F-measure, and the Youden J statistic. For use in precision–recall curves, the F-score was used, defined as:

(3) FScore=(2∗Precision∗Recall)∕(Precision+Recall)

A series of decision thresholds can be used to fit a training data set, and the classifier parameters from the training data applied to the test set. The F score metric is then computed for each test set.. The maximum value of the F-score was assigned as the ‘best’ value and the corresponding decision threshold was assigned as the optimal value. Accuracy can also be computed as the number of correct predictions divided by the total number of predictions, or (TP + TN)/(TP + TN + FP + FN). However, when dealing with class-imbalanced data, there is a significant disparity between the number of positive and negative class members. Hence, for negative members, there is a much higher probability of correct prediction and the true negatives dominate the accuracy parameter, giving a misleading view of model effectiveness [29]. Thus, accuracy was not used as a measure of model capability.

**Figure 2.**
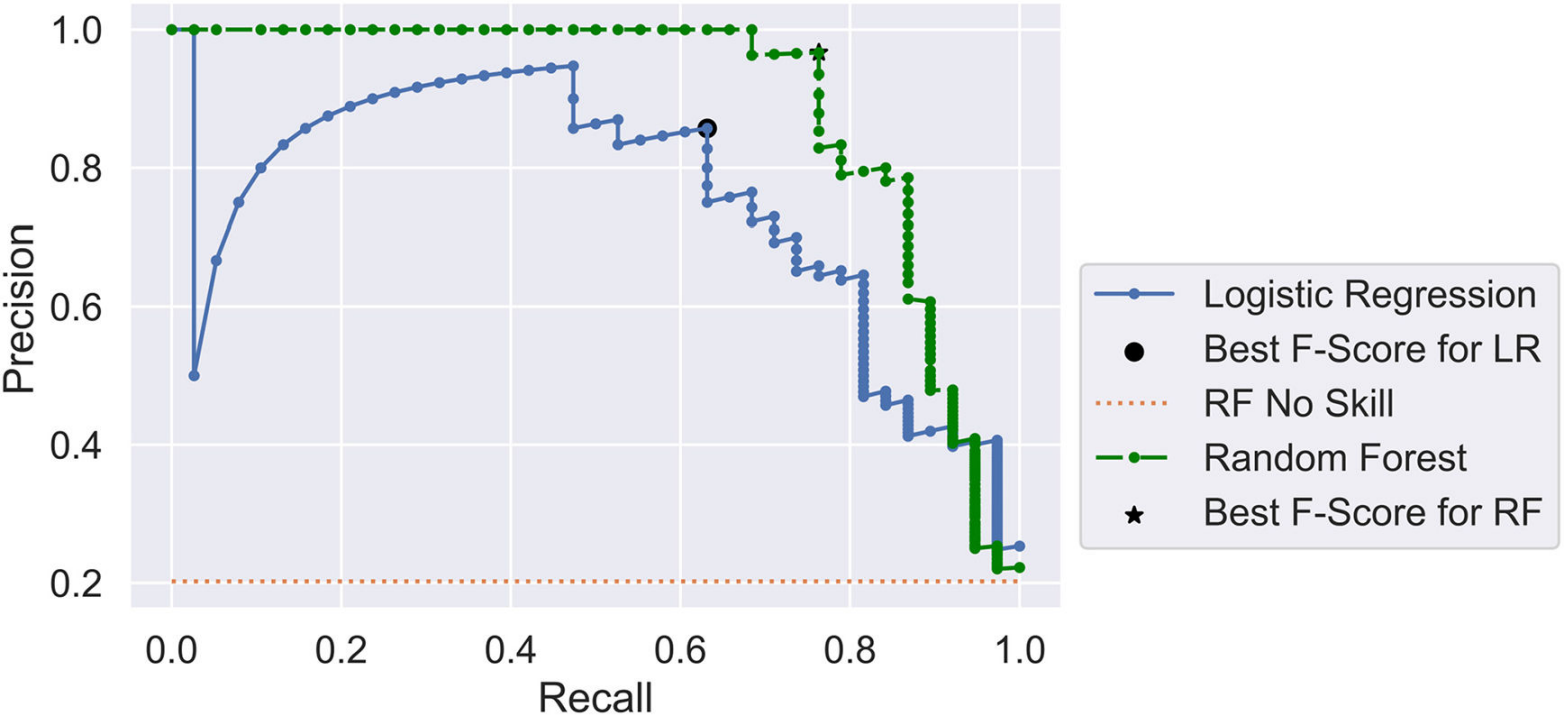
Precision–recall curves for both a random forest and a logistic regression model of the Lawson Prime data set using 18 features. The black dot and star show the precision–recall points for the best F-score after threshold tuning on the logistic regression and random forest models, respectively. The orange dotted line shows what performance would be expected if the model had no classification skill.

Along with the random forest analysis, results from a logistic regression were included for comparison purposes. In both cases, 468 points from the Lawson Prime data were used, with a 60/40 split between the training and test data. Results are shown for the test data. Default values in the Sklearn Python library [15] were used in both models, with the exception that 2000 trees were used in the random forest model. Overall, the random forest model performed better than the logistic regression. Figure 2 shows a graphic presentation of the precision–recall curves from the two models. A perfect classifier would have a point in the upper right-hand corner of the plot. The random forest classifier (shown in green) consistently scored higher than the logistic regression (shown in blue). The random forest classifier had a precision of 96.7% at its highest F-score, meaning that 96.7% of the points in the minority positive class were classed correctly. Similarly, a recall score of 76.3% indicates that 76.3% of the total number of dynamic failure cases was correctly classed.

Sklearn also allows a measure of the importance of each feature used in the random forest model to be computed. In Sklearn, the Gini importance [30] is used as this measure. Importance levels are normalized so the sum over all features goes to 1. The features with importance measures greater than 0.05 are oxygen, pyritic sulfur, volatile matter, the Van Krevelen ratio, organic sulfur, moisture, and vitrinite reflectance. For the most part, these features are the same as those found in all the other analyses, indicating the consistency in critical feature selection.

**Figure 3.**
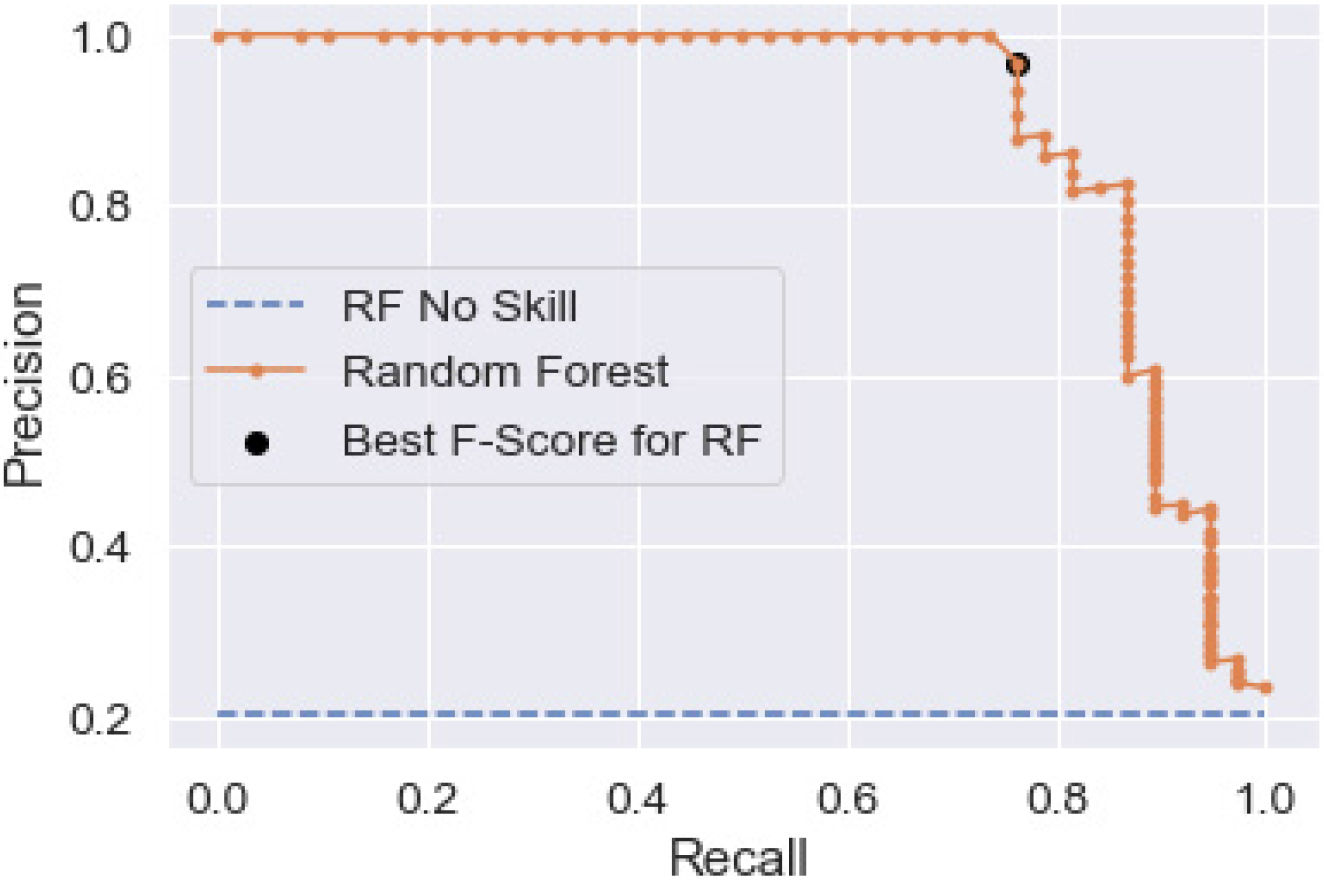
Precision–recall curve for Lawson Prime data after completion of 5-fold cross-validation. The ‘No-Skill’ line and the best precision–recall point defined by the maximum F-score are shown.

Finally, although the threshold was tuned in the results just shown, the default algorithm values used in the random forest model may not be optimal for model building. To examine this, a feature search using cross-validation was performed. This was completed through the use of a randomized grid search of parameters using 5-fold cross-validation. A total of 500 different combinations were examined. The best parameters obtained were then used to run another random forest and the results evaluated as before. The parameters found in the search, which were different from the default values used previously, were: the number of trees = 1800, the minimum samples per split = 5, and the minimum samples per leaf = 2. The precision–recall plot for these values, again using a 60/40 training–test split, is shown in Figure 3. The ‘best’ point on the precision–recall curve, as defined by the maximum F-score, is also shown.

Table 6 provides the threshold, precision, and recall values for the model after 5-fold cross-validation and threshold tuning. The optimal threshold is slightly different, but precision and recall show no change. Thus, while cross-validation is probably a desirable processing step to ensure that the data are not overfitted, overall model capability is not different from simple threshold tuning.

Because the majority of dynamic failure cases come from the western U.S. and the majority of control cases from eastern coal fields, it could be argued that simply dividing the data between states of occurrence would produce equal or better results than a machine learning model. This sort of model was constructed by placing all eastern coal points in a control class and all western coals in the dynamic failure class. The result of this very simple model was a precision score of 86.6% and a recall score of 78.8%. Thus, simply classing the occurrences by state yields a good independent classifier. However, these results are not as good as those from a random forest model, nor do they explain any potential risk factors. A summary of precision and recall for the various models is shown in Table 7.

In summary, a random forest classifier appears to provide superior performance over logistic regression, as measured by precision and recall values. Tuning the decision threshold provides the same level of model improvement as performing a cross-validation on the features, although the cross-validation may provide some protection from overfitting. In operating on the class-imbalanced Lawson Prime data set, the random forest model achieves a precision score of 96.7% and a recall score of 76.3%. In other words, of the events that were classed as dynamic failure seams by the model, 96.7% actually experienced dynamic failures. Of the total number of bump-positive events, 76.3% were correctly classed.

### 2.4. Cluster Analysis and Dimensionality Reduction

Another objective of the current study was to explore possible relationships between features measurable in coals with respect to the occurrence of dynamic failures. This was carried out using cluster analysis to explore possible groupings in the data based on the feature properties.

Several types of cluster analyses are available in statistical software packages. In this study, the hierarchical density-based spatial clustering of applications with the noise (HDBscan) algorithm was used [13,31,32]. HDBscan offers several advantages over other clustering algorithms. In line with other true clustering algorithms, HDBscan delineates density-based clusters as opposed to partitions. It does not require spherical or elliptical groupings for good performance; it does not require any specification of the number of clusters; and it has the ability to omit single points, noise, and outliers from the cluster membership.

The number of features used was reduced to the top seven, as determined from random forest analysis. These were: oxygen, pyritic sulfur, the Van Krevelen ratio, volatile matter, moisture, organic sulfur, and vitrinite reflectance. As few as four features were analyzed, but the results were not dramatically different from the results using seven features.

As with logistic regression analysis, feature values were scaled using a centering algorithm. Scaling preserves the shape of the original distribution and does not significantly change the information embedded in the original data [33], nor does it reduce the importance of outliers. With the seven features listed above, using a minimum cluster size of 10 points and a minimum number of 3 samples, HDBscan found three clusters. The minimum cluster size is an intuitive parameter as it defines the smallest size grouping in order to be considered a cluster. Minimum samples are less intuitive, but broadly speaking, they determine how conservative clustering is. The larger the value, the more points that will be declared as noise, and clusters will be restricted to progressively denser areas [13].

**Figure 4.**
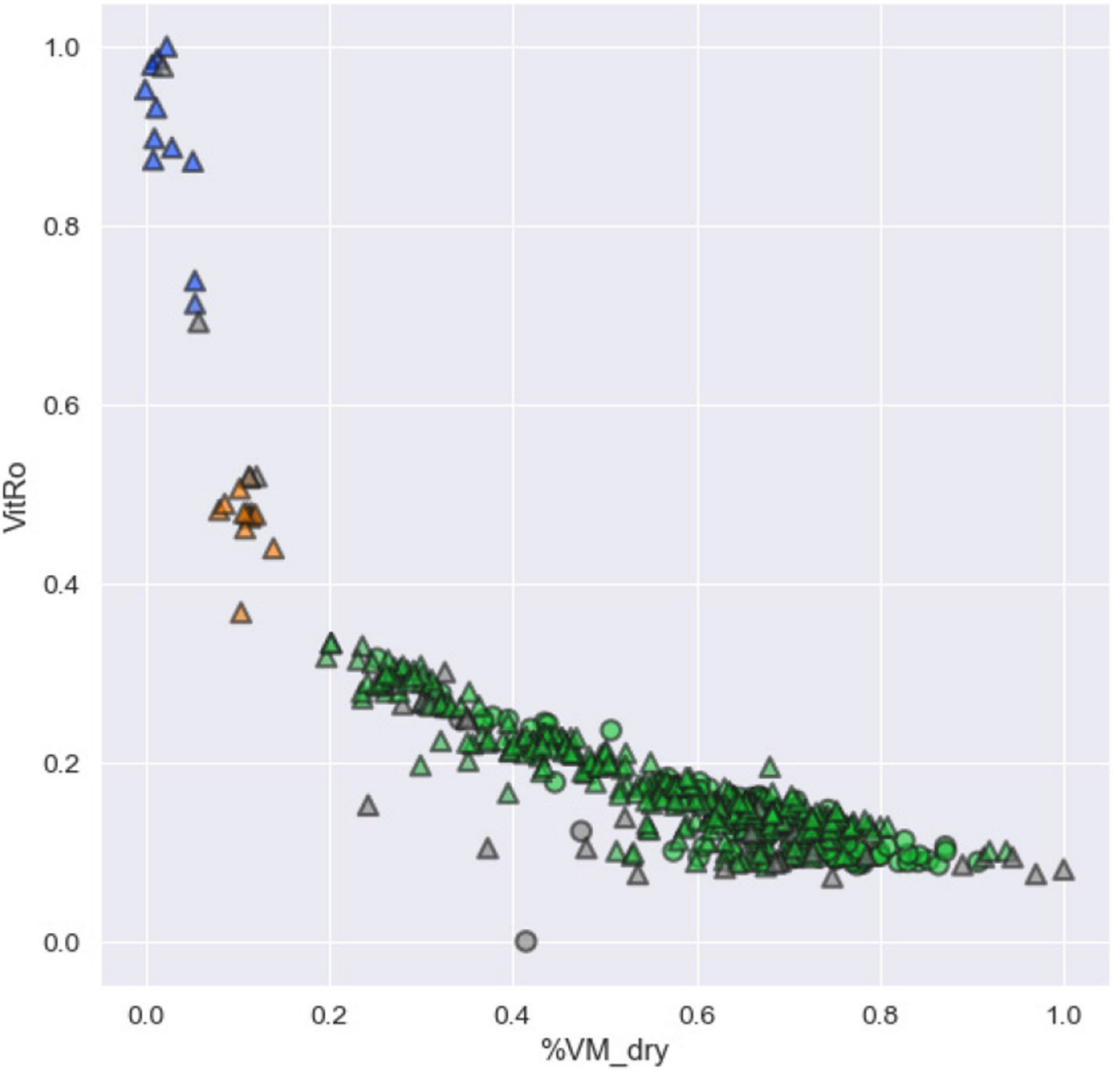
Clustering results from HDBscan when using the top seven features, as determined by random forest analysis after 5-fold cross-validation. This two-dimensional cross-plot shows three clusters found with HDBscan in different colors. The clusters are distinct, but do not distinguish dynamic failure from non-failure coals.

From the seven-feature clustering, a representative cross-plot of the clusters is shown in Figure 4. This plot is a two-dimensional slice through a seven-dimensional space. Here, the three clusters found by HDBscan are shown in blue, orange, and green. The points in grey were considered outliers and were not assigned to a cluster. The triangles denote the points from control seams and the circles denote the dynamic failure seams, as determined in the initial data selection. The three clusters are relatively well separated but do not separate the dynamic failure and control group seams.

The main objective of this clustering exercise was to see if an unsupervised machine learning technique could separate the dynamic failure and control coal members, without any additional processing or operator interpretation. While some interesting relations were seen, this approach did not appear to be successful in discrimination between the two classes. One reason the classes could not be discriminated may be the number of dimensions present in the data and the complexity of the resulting cluster analysis. A means to explore a simpler data structure was pursued through dimensionality reduction.

Although the Lawson Prime data set can be considered to be of limited size, both in terms of the number of members and features, even this limited dimensional data can be difficult to visualize and extract useful information. As data dimensions increase, the ‘curse of dimensionality’ [34] arises, and model performance suffers. Sample density decreases exponentially as dimensions increase, and eventually the concept of distance between points, as used in cluster analysis, becomes undefined. One way of overcoming the curse of dimensionality that arises with increasing features and limited training points is to employ dimensionality reduction to project data into lower dimensions. In addition, projections of a higher-dimension sample space onto lower dimensions may reveal structures in the data that are not otherwise apparent.

There are several approaches that can be used to perform dimension reduction, with the most common being principal component analysis (PCA). PCA assumes linear relations between features and works to rotate and project data along directions of increasing variance. PCA can be very successful when applied to data with linear relationships, but fails when the data do not lie on a linear subspace.

At this stage of analysis on the Lawson Prime data set, it was not clear that features can be represented as linear combinations of each other, as is assumed in PCA. Thus, both a linear (PCA) and a nonlinear dimensionality reduction algorithm were employed to determine which provided more interpretable results. The nonlinear dimensionality reduction algorithm applied was t-distributed stochastic neighbor embedding (t-SNE). Briefly, t-SNE computes the probability that pairs of points in a higher-dimensional space are related and computes a lower-dimension embedding that attempts to preserve that distribution [14,35-37]. The main advantages of t-SNE are that it can be applied to nonlinear data and can preserve local and global data structures. Unfortunately, it is computationally expensive and is nondeterministic.

PCA analysis was performed with the same seven features, retaining the top four principal components. The variance ratios explained by the first four principal components are given in Table 8. The top two components explained 75.6% of the variance, the top three explained 85.4%, and the top four explained 93.2%.

**Figure 5.**
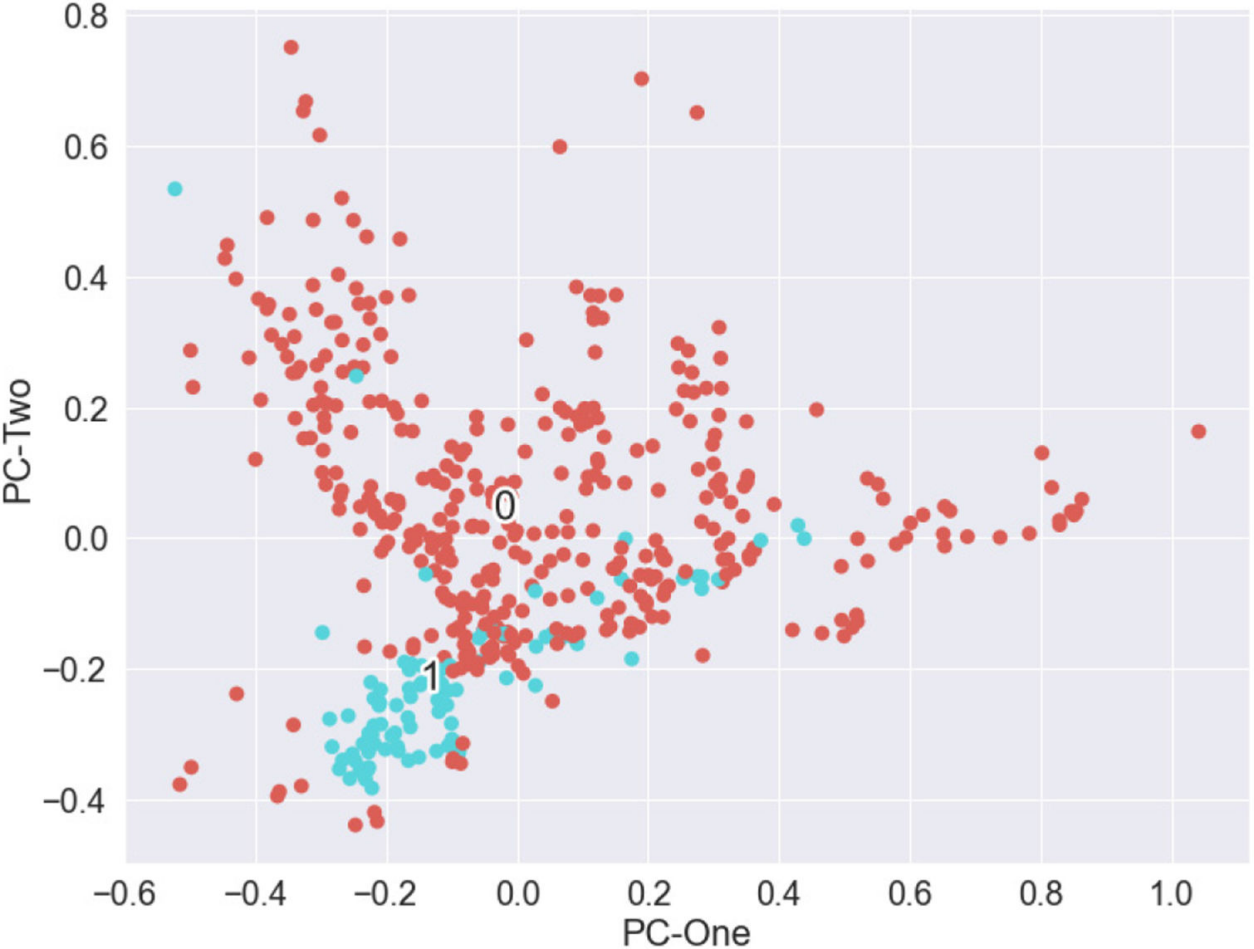
Plot of the two-dimensional principal components for the Lawson Prime data set. Blue points referenced with ‘1’ signify dynamic failure coal members. Brown points with ‘0’ are the majority class control members.

Figure 5 shows the scaled Lawson data points projected onto the top two principal components, PCA1 and PCA2, showing the results of reducing the feature dimensions from 7 to 2. In this figure, the labels ‘0’ (control group, brown) and ‘1’ (dynamic failure group, blue) were plotted at the centroid of the respective point clouds. The bumping and non-bumping points were clustered with like members. However, while there was some separation between the classes, it may not be sufficient to identify them as distinct groups without a priori information of the membership class. Dynamic failure cases were assembled at the smallest values of components 1 and 2.

To examine the impact of each of the seven retained features on the four principal components, both an impact table and a biplot were produced [38,39]. From this analysis, it was found that percent volatile matter dominated PC1. Pyritic sulfur exerted a strong positive impact on PC2, and organic sulfur had a strong positive impact on PC3.

While performing PCA on the Lawson data did show some separation between dynamic failure and control data, the separation was not sufficient to allow distinct clusters to be identified without a priori information. t-SNE was applied to the same data set to search for identifiable structures in the data. Algorithm parameters used were the number of iterations = 5000, the learning rate = 20, early exaggeration = 10, perplexity = 25 and metric = ‘Manhattan’. When using a larger number of dimensions, several authors recommend against the use of an L_2_ norm (Euclidean distance) [34], and so a ‘Manhattan’ metric or L_1_ norm was used. Perplexity provides a general measure of the structure of the data, with larger values emphasizing more global structures, as opposed to local variations. Larger values of perplexity can be thought of as using more neighbors in the calculation. Wattenberg et al. [40] recommend that the perplexity should not be set much greater than the number of data points and to tailor the parameters only to show possible structure in the data set, as opposed to interpreting shapes and distances between clusters.

**Figure 6.**
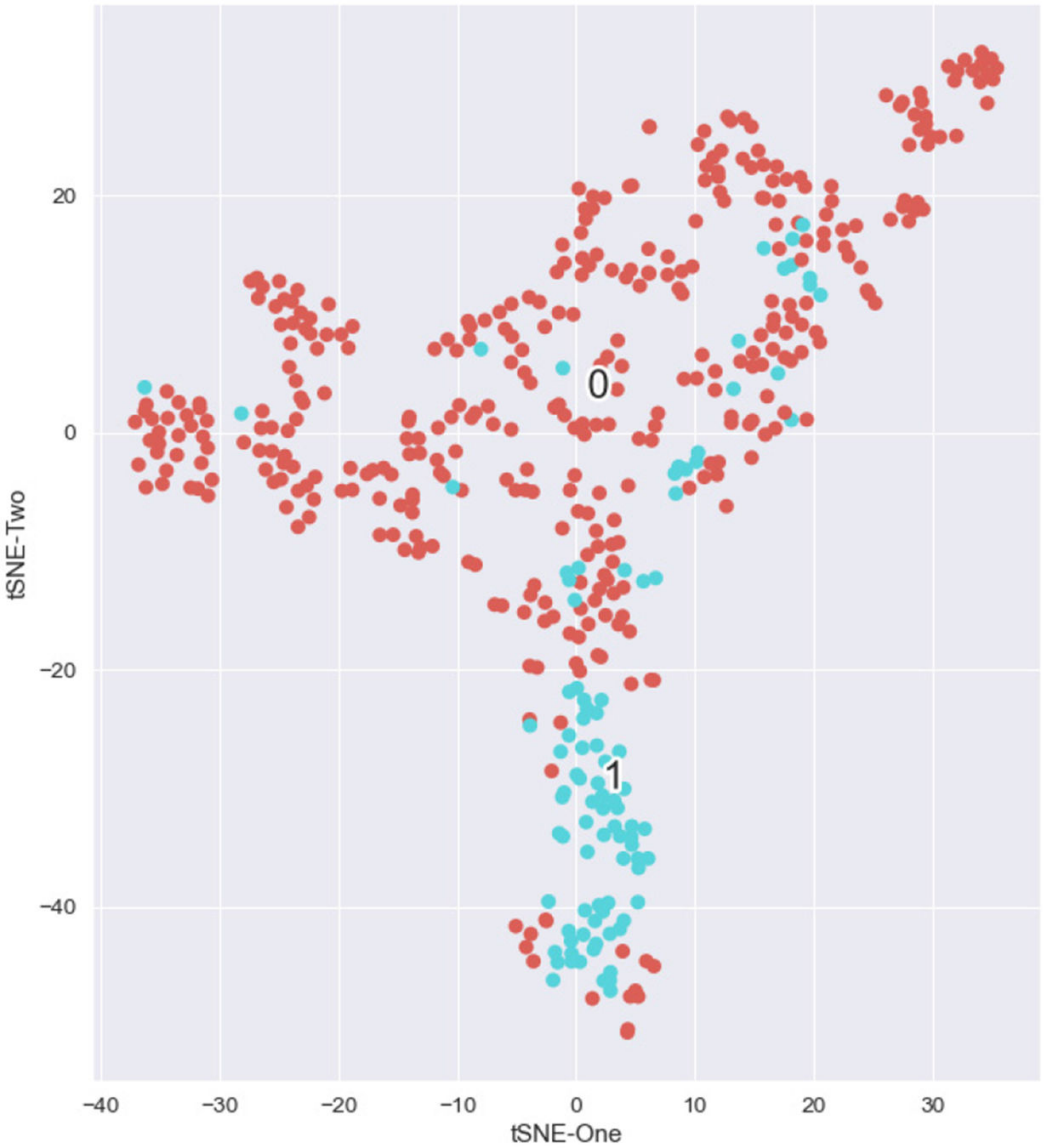
t-SNE results where seven dimensions were reduced to two for the Lawson Prime data set. In general, the bumping points (‘1’, blue) were well separated from the non-bumping (‘0’, orange).

Figure 6 shows t-SNE results when processing the scaled 7-dimensional data. Retaining two components from t-SNE showed improved separation between clusters. It was found that two major clusters exist in the data: one of nearly exclusive control points and one of mostly dynamic failure data points. There is an interesting structure to this plot, as it appeared that the control coals are mostly sensitive to tSNE1 and the bumping coals are mostly sensitive to t-SNE2. These possible dependencies need further investigation. There were several bumping data points scattered throughout the non-bumping cluster, along with bumping points in the non-bumping group, which require further investigation. The majority of bumping points are clustered in a group at the bottom of Figure 6.

**Figure 7.**
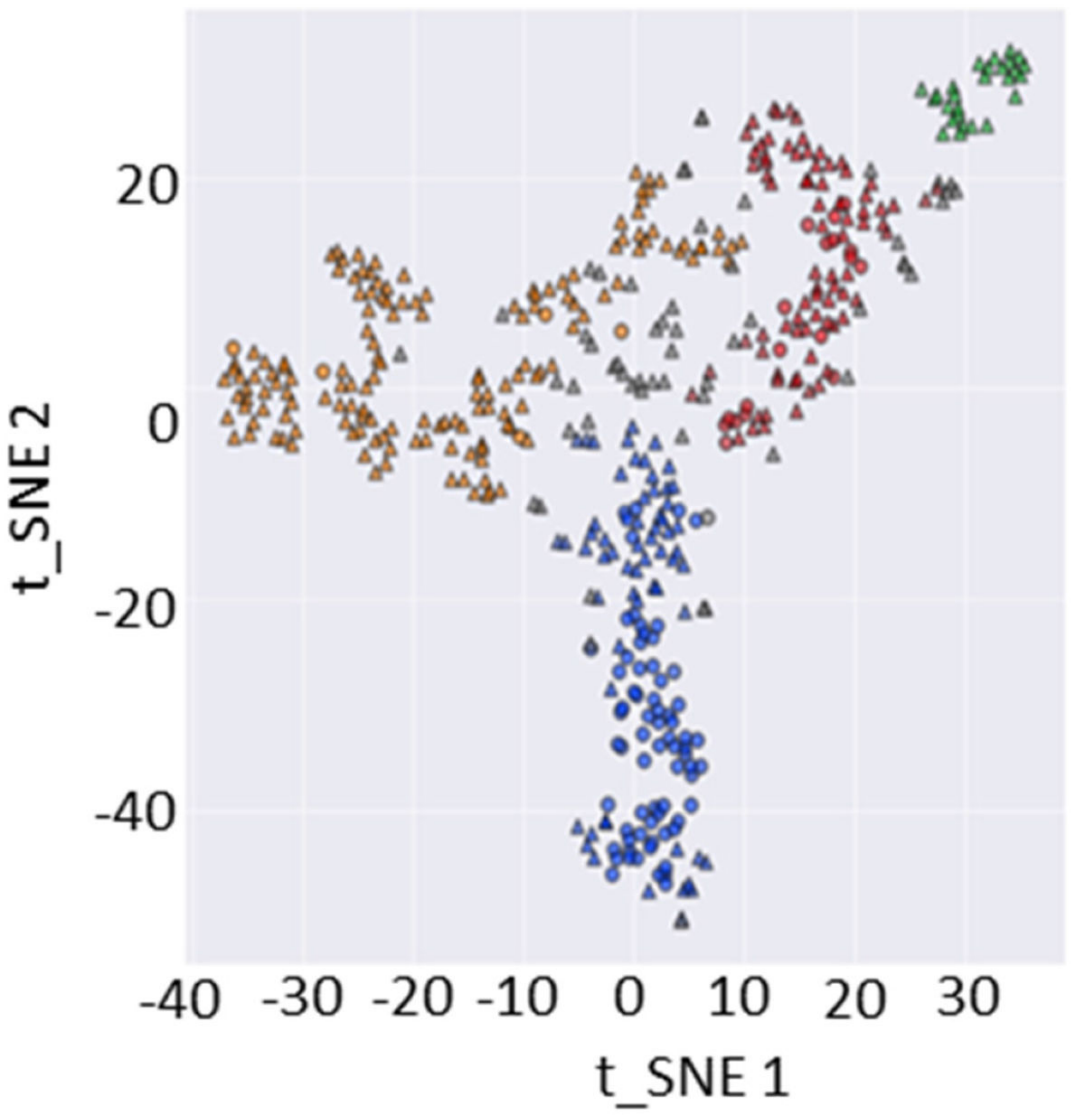
Plot of the t-SNE-processed data after the application of HDBscan. Four clusters—green, red, orange, and blue—were found. The blue cluster is largely the dynamic failure coal data.

Preliminary analysis was completed to examine if HDBscan could independently find control and dynamic failure clusters from the t-SNE-processed data. The same clustering parameter values used previously were applied to the two-dimensional t-SNE values, as plotted in Figure 6. Figure 7 shows the results of this cluster analysis.

As in other plots, triangles and circles indicate non-bumping and bumping coals, respectively. HDBscan computed four clusters in the data, as shown in Figure 7.

In the two component t-SNE results of Figure 6, a number of points from control seams were located among dynamic failure points, and vice versa. A total of 17 control data points were located in the area of dynamic failure points. In addition, 29 dynamic failure points were scattered throughout the control population. Within all measurements, noise may be present which could cause these points to be located within groups opposite from their ground truth. Similarly, these data may be outliers that do not follow the general behavior seen on most points of either population. However, it is more likely that this may reflect the impact, or lack thereof, of mining-induced stresses and other in situ, real-world variables that impact dynamic failure occurrence.

To further examine the potential reasons for this structure, each of the points was identified in the original 18 dimensional feature space and properties examined. First, the data points belonging to surface mines were identified and removed from consideration. This removed 9 points from the control population that were plotted among the dynamic failure coal points and 5 surface mines from the complementary population. Thus, the number of apparently anomalous data points was reduced by 14. The remaining points were examined in terms of the mines from which they were taken, geologic setting, and overall geochemical signatures.

The most critical question to be addressed in these data concerns why dynamic failure points are plotted in the control group. This is a false-negative case and bears the highest cost in terms of potential risk analysis. The distribution of the remaining 24 points in this group with a state and basin distribution is shown in Table 9 (as determined from entries in the PSOC database).

In this group, 17 points were taken from the Appalachian Basin and 7 were taken from the Uinta-Piceance Basin. A more detailed look at the source of the 24 data points showed that two were reported from an eastern mine that failed while mining took place over unknown barrier pillars in a lower mined seam. The cause of the bumps was attributed to this anomalous stress distribution. Six of the points came from the deep gassy L.S. Wood #3 mine in Colorado, and may be the result of gas-driven failure. One sample had geochemical values that matched control coals, but was from a mine under unusually deep cover. This loading may have been the critical factor in the failure. Four points were plotted close to the boundary of the dynamic failure group, and so they may be simple outliers to that population. Six had geochemical values that matched the averages for control coals but had pyritic sulfur ranges that were closer to the dynamic failure averages. Finally, five matched the control group values, and no further mine information could be found.

The second group of anomalous points was plotted closer to the dynamic failure group data, even though they were not reported as having experienced a reportable event. Four of these came from the Cannel or King Cannel seams in Utah. No information could be found on the source mines. These four points had geochemical values closer to the dynamic failure group coals, but experienced no reportable events. Further investigation is needed to determine if these were simply too shallow to experience dynamic failure or if they lack other necessary contributing factors to produce dynamic failure occurrence. The last four points were mixed in terms of their composition as regards the average of dynamic failure coals. It is not clear why these coals plot as they do, although two were taken from the Lower Kittanning seam. All points from this seam had property values well in the control range, except for these two which may represent statistical outliers.

The average values for the features used in t-SNE for dynamic failure, control, and the two anomalous classes are shown in Table 9. In Table 9, the feature values for the control points that plot in the dynamic failure cluster match the general values for dynamic failure coals, and vice versa. The exception to this was based on the sulfur content.

For the seven highest ranked random forest features, there were no significant differences between the ‘anomalous’ data points and the majority of points in that group. For example, the organic and pyritic sulfur did not show consistent differences between the four groups. However, oxygen, percent volatile matter, Van Krevelen ratio, percent moisture, and vitrinite reflectance features showed a consistent correlation. For all these features, the average values of the ‘Bump in Bump group’ and the ‘No-Bump in Bump group’ were similar in value. Likewise, the ‘No-Bump in No-Bump group’ and the ‘Bump in No-Bump group’ were similar (Table 10). Thus, it appears that even though they did not fail dynamically, the control points in the dynamic failure group had geochemical markers more similar to the dynamic failure coals. Other parameters thus need to be examined for these coals. Likewise, the coals that experienced dynamic failure but appeared to be in the control group had feature values that were more similar to the bulk of the control coals. This echoes the findings of Babcock and Bickel [41] which exhibit that nearly any coal can be induced to dynamically fail under the appropriate conditions. However, some may do so with relatively greater ease. These coals all need to be studied in more detail in order to determine why they have geochemical values that match the opposite population. Additionally, more studies are greatly needed to determine if the presence (or dearth) of certain chemical species contributes to dynamic failure on a fundamental level.

## 3. Summary

The objectives of this study were: (1) to determine if machine learning could be applied to a larger data set than previously attempted, (2) to apply cluster analysis and dimensionality reduction to look for previously unnoticed data structure, and (3) to explore if machine learning could provide an accurate model to discriminate coals that experienced dynamic failures based on geochemical signatures. Based on analyses summarized in this study and prior works, it appears that there is at least an empirical correlation between coal compositional features and dynamic failure occurrence in an in situ mining environment. However, it is not clear what the underlying cause of this correlation is; whether the compositional attributes are proxies for other geologic factors, they exert a fundamental effect on coal physical properties, or the observed relation is coincidental. Recent work carried out by Kim et al. [4] suggests that mineral content may exert some impact on the intrinsic ability of coal to retain energy prior to failure, and that this may differ depending upon the degree of cleat development. It may be possible that features such as vitrinite reflectance and the Van Krevelen ratio serve as proxies for cleat development, which is an attribute that was not directly measured in this study.

Logistic regression performed on the Lawson data determined that 6 features of the 18 that we examined exerted the biggest impact on the results. These features were pyritic sulfur, organic sulfur, oxygen, percent volatile matter, percent ash, and vitrinite reflectance. These features were used to build a model and 3000 trials were performed to account for the relative sparsity of the dynamic failure cases; the average recall of the model was 81.9%. Thus, the regression model correctly classified the data points approximately 82% of the time.

A comparison was made for both logistic regression and random forest classifiers where a default threshold of 0.5 and a tuned threshold were applied. The random forest classifier performed better than the logistic regression in both cases, achieving a precision of 96.7% in the tuned threshold case.

Cluster analysis was performed using the top seven features determined with the random forest classifier. The application of HDBscan resulted in three clusters being found in the scaled data. The significance of these clusters is not clear. The dynamic failure samples showed some separation in the two-dimensional projection of seven-dimensional data, but not enough to allow automated separation into their own cluster.

Both linear (PCA) and nonlinear (t-SNE) dimensionality reduction operations were applied to the data. Again, the top seven features determined from the random forest classifier were used. In two dimensions, the PCA plot showed a mass of bumping points at small values of PCA1 and PCA2, corresponding to small pyritic and organic sulfur content and small volatile matter values. This aligns with prior work.

The application of t-SNE to the Lawson set provided a better separation of the dynamic failure and control data in two dimensions. The dynamic failure cases formed a relatively distinct grouping, although some points were dispersed through the control data cloud. Cluster analysis on t-SNE-processed data resulted in the HDBscan finding four clusters, with one made up of mostly dynamic failure data and the other three predominantly made up of the control group.

Finally, an explanation of why some dynamic failure coals were plotted with control coals, and vice versa, is needed. As a blanket statement, it appears that this mixture is due to additional confounding factors. For dynamic failure samples scattered in the control group shown in the t-SNE plot of Figure 6, 19 of 24 samples could be at least partially explained by other confounding factors. For the complementary set, four points were taken from a Utah coal mine which compositionally matched dynamic failure coals. However, these samples did not suffer dynamic failures. Information regarding the mines from which these samples originated could not be found in the MSHA data retrieval system or from extensive internet searches.

In conclusion, a successful machine learning classifier, with 96.7% precision and 76.3% recall rates in classifying coals pertaining to whether they experienced dynamic failures or not, based on geochemical data, was constructed. A simple classifier based on the state of origin did not perform as well as a random forest, although the state model worked surprisingly well. Dimensionality reduction using t-SNE revealed structure in the data that offered opportunities to investigate what appear to be anomalous data points.

## Figures and Tables

**Table 1.** Distribution of data samples by state of occurrence.

Distribution of Seam Data by State								
State	PA	WV	KY	UT	CO	AL	TN	IL
Number of Data Points	191	108	66	55	36	5	3	4

**Table 2.** Distribution of data samples by coal rank.

Coal Rank	Number of Data Points
Anthracite	15
Semi-anthracite	12
Low-volatility bituminous	54
Medium-volatility bituminous	73
High-volatility bituminous A	215
High-volatility bituminous B	57
High-volatility bituminous C	38
Sub-bituminous A	3
Lignite	1

**Table 3.** Features retained from the Lawson Prime data set used in RFECV.

Feature Numbers	Features Used in RFECV Analysis
1	% Moisture (as-received)
2	% Ash (dry)
3	% Volatile matter (dry)
4	% Carbon (dry, ash free)
5	% Hydrogen (dry, ash-free)
6	% Nitrogen (dry, ash-free)
7	% Organic sulfur (dry)
8	% Oxygen (diff)
9	Carbon-nitrogen ratio (dry, ash-free)
10	Hydrogen–carbon ratio (dry, ash-free)
11	Van Krevelen ratio (dry, ash-free)
12	% Pyritic sulfur (dry)
13	% Vitrinite (mineral-matter-free)
14	% Liptinite (mineral-matter-free)
15	% Inertinite (mineral-matter-free)
16	% Vitrinite reflectance
17	Oxygen–inertinite ratio
18	Vitrinite–(inertinite + liptinite) ratio

**Table 4.** Confusion matrix for logistic regression classifiers showing average values.

		Actual Values	
		T = 1	F = 0
Classifier Results	T = 1	32.8	6.5
	F = 0	7.2	25.5
	Total	40	32

**Table 5.** Average precision, recall, and F-score for test classifiers.

	Average Value	Standard Deviation
Average precision	0.812	0.034
Average recall	0.810	0.032
Average F-score	0.810	0.033

**Table 6.** Random forest performance after 5-fold cross-validation and threshold tuning.

Random Forest Model after 5-Fold Cross-Validation	
Tuned threshold	0.492
Maximum F-score	0.853
Best model precision	96.7%
Best model recall	76.3%

**Table 7.** Precision and recall results for the tested classifiers.

	Precision	Recall
Logistic regression down-sampled majority class	81.2%	81.0%
Logistic regression default threshold	78.9%	63.1%
Logistic-regression-tuned threshold	85.7%	63.2%
Random forest default threshold	96.5%	76.3%
Random-forest-tuned threshold	96.7%	76.3%
Random-forest-tuned hyperparameters	96.7%	76.3%
Simple state classifier	86.6%	78.8%

**Table 8.** Variance ratios explained by the first four principal components for the Lawson Prime data set.

Principal Components	Variance Ratios Explained
1	0.463
2	0.293
3	0.098
4	0.078

**Table 9.** State and basin distribution of dynamic failure data plotting in the control group.

		State Distribution	
Kentucky	West Virginia	Colorado	Utah
8	9	6	1
		Basin Distribution	
Appalachian	Uinta-Piceance		
17	7		

**Table 10.** Average feature values for the data populations, with surface mines removed.

	Pyritic Sulfur	Organic Sulfur	% Moisture (as-Received)	% Volatile Matter (Dry)	Oxygen	Van Krevelen Ratio	Vitrinite Reflectance (%)
Bump in non-bump group	0.144	0.558	1.479	28.80	5.479	20.43	1.209
Non-bump in bump group	0.386	0.639	7.315	39.48	13.17	9.949	0.744
Bump points	0.130	0.482	5.514	40.36	12.23	8.106	0.627
Non-bump points	1.268	0.876	2.486	29.14	6.071	16.75	1.252

## Data Availability

MSHA accident and fatality data are available at www.msha.gov. For information regarding the PennState Coal Sample Databank, please visit https://www.energy.psu.edu/facilities/penn-state-coal-sample-bank (accessed on 30 January 2019).

## References

[1] Lawson H; Weakley A; Miller A Dynamic failure in coal seams: Implications of coal composition for bump susceptibility. Int. J. Min. Sci. Technol 2016, 26, 3–8.

[2] Berry C; Warren S; Hanson D Investigating the Correlation Between Coal Geochemistry and Coal Bumps. In Proceedings of the 38th International Conference on Ground Control in Mining, Morgantown, WV, USA, 23–25 July 2019; Society for Mining, Metallurgy & Exploration: Englewood, CO, USA, 2019; p. 171.

[3] Lawson H Exploration of petrographic, elemental, and material properties of dynamic failure-prone coals. Int. J. Min. Sci. Technol 2020, 30, 69–75. 32685238 10.1016/j.ijmst.2019.12.015 PMC7367612

[4] Kim BH; Larson MK; Lawson H; Walton G Influence of Mineralogical Compositions on Anisotropic Burst-Prone Coal Strength. In Proceedings of the International Conference on Ground Control in Mining, Canonsburg, PA, USA, 26–28 July 2022.

[5] Mathews JP; Krishnamoorthy V; Louw E; Tchapda A; Castro-Marcano F; Karri V; Alexis DA; Mitchell GD A review of the correlations of coal properties with elemental composition. Fuel Process. Technol 2014, 121, 104–113.

[6] McGaughey J Artificial Intelligence and Big Data Analytics in Mining Geomechanics. In Deep Mining 2019: Proceedings of the Ninth International Conference on Deep and High Stress Mining; Joughin W, Ed.; The Southern African Institute of Mining and Metallurgy: Johannesburg, South Africa, 2019; pp. 45–54. Available online: 10.36487/ACG_rep/1952_04_McGaughey (accessed on 15 June 2021).

[7] McGaughey W; Lafleche V; Howlett C; Sydor J; Campos D; Purchase J; Huynh S Automated, real-time geohazard assessment in deep underground mines. In Proceedings of the Eighth International Conference on Deep and High Stress Mining; Wesseloo J, Ed.; Australian Centre for Geomechanics: Perth, Australia, 2017; pp. 521–528.

[8] Li Y; Wang C; Liu Y Classification of Coal Bursting Liability Based on Support Vector Machine and Imbalanced Sample Set. Minerals 2022, 13, 15.

[9] Wojtecki L; Iwaszenko S; Apel D; Cichy T An attempt to use machine learning algorithms to estimate the rockburst hazard in underground excavations of hard coal mine. Energies 2021, 14, 6928.

[10] Pu Y; Apel D; Liu V; Mitri H Machine learning methods for rockburst prediction-state-of-the-art review. Int. J. Min. Sci. Technol 2019, 29, 565–570.

[11] Li Z; Wang E; Ou J; Liu Z Hazard evaluation of coal and gas outbursts in a coal-mine roadway based on logistic regression model. Int. J. Rock Mech. Min. Sci 2015, 80, 185–195.

[12] Mark C; (MSHA, Pittsburgh, PA, USA). Personal communication, 2020.

[13] McInnes L; Healy J; Astels S HDBscan: Hierarchical density-based clustering. J. Open Source Softw 2017, 2, 205.

[14] Van der Maaten L; Hinton G Visualizing data using t-SNE. J. Mach. Learn. Res 2008, 9, 2579–2605.

[15] Pedregosa F; Varoquaux G; Gramfort A; Michel V; Thirion B; Grisel O; Blondel M Scikit-learn: Machine learning in Python. J. Mach. Learn. Res 2011, 12, 2825–2830.

[16] Browne-Anderson H Preprocessing in Data Science (Part 2): Centering, Scaling and Logistic Regression. 2016. Available online: https://www.datacamp.com/community/tutorials/preprocessing-in-data-science-part-2-centering-scaling-and-logistic-regression (accessed on 1 May 2020).

[17] Misra P; Yadav AS Improving the classification accuracy using recursive feature elimination with cross-validation. Int. J. Emerg. Technol 2020, 11, 659–665.

[18] Gupta P Regularization in Machine Learning. 2017. Available online: https://towardsdatascience.com/regularization-in-machine-learning-76441ddcf99a (accessed on 15 May 2020).

[19] Witten JG; Hastie D; Tibshirani R An Introduction to Statistical Learning; Springer: New York, NY, USA, 2013; p. 112.

[20] King G; Zeng L Explaining rare events in international relations. Int. Organ 2001, 55, 693–715.

[21] King G; Zeng L Logistic regression in rare events data. Polit. Anal 2001, 9, 137–163.

[22] Greenland S; Schwartzbaum J; Finkle W Problems due to small samples and sparse data in conditional logistic regression analysis. Am. J. Epidemiol 2000, 151, 531–539. 10707923 10.1093/oxfordjournals.aje.a010240

[23] Ruiz A; Villa N Storms prediction: Logistic regression vs random forest for unbalanced data. arXiv 2018, arXiv:0804.0650.

[24] Zhang H; Bi Y; Jiang W; Cao S; Guo P; Zhang J Application of random forest classifier in loan default forecast. In International Conference on Artificial Intelligence and Security; Springer: Singapore, 2020; pp. 410–420.

[25] Brownlee J A Gentle Introduction to Threshold-Moving for Imbalanced Classification. 2021. Available online: https://machinelearningmastery.com/threshold-moving-for-imbalanced-classification/ (accessed on 15 August 2020).

[26] Provost F Machine learning from imbalanced data sets 101. In Proceedings of the AAAI’2000 Workshop on Imbalanced Data Sets; AAAI Press: Washington, DC, USA, 2000; Volume 68, pp. 1–3.

[27] Brownlee J How to Grid Search Hyperparameters for Deep Learning Models in Python with Keras. 2016. Available online: https://machinelearningmastery.com/grid-search-hyperparameters-deep-learning-models-python-keras (accessed on 15 August 2022).

[28] Koehrsen W Beyond Accuracy: Precision and Recall. 2018. Available online: https://towardsdatascience.com/beyond-accuracy-precision-and-recall-3da06bea9f6c (accessed on 15 May 2020).

[29] Brownlee J Failure of Classification Accuracy for Imbalanced Class Distributions. 2020. Available online: https://machinelearningmastery.com/failure-of-accuracy-for-imbalanced-class-distributions/ (accessed on 15 June 2021).

[30] Zhou V A Simple Explanation of Gini Impurity. 2019. Available online: https://victorzhou.com/blog/gini-impurity/ (accessed on 15 May 2020).

[31] Campello R; Moulavi D; Sander J Density-based clustering based on hierarchical density estimates. In Pacific-Asia Conference on Knowledge Discovery and Data Mining; Springer: Berlin/Heidelberg, Germany, 2013; pp. 160–172.

[32] McInnes L; Healy J Accelerated hierarchical density based clustering. In Proceedings of the 2017 IEEE International Conference on Data Mining Workshops (ICDMW), New Orleans, LA, USA, 18–21 November 2017; pp. 33–42.

[33] De Souto M; De Araujo D; Costa I; Soares R; Ludermir T; Schliep A Comparative study on normalization procedures for cluster analysis of gene expression datasets. In Proceedings of the 2008 IEEE International Joint Conference on Neural Networks (IEEE World Congress on Computational Intelligence, Hong Kong, China, 1–8 June 2008; pp. 2792–2798.

[34] Aggarwal C; Hinneburg A; Keim D On the surprising behavior of distance metrics in high dimensional space. In International Conference on Database Theory; Springer: Berlin/Heidelberg, Germany, 2001; pp. 420–434.

[35] Van der Maaten L Visualizing Data Using Embeddings. 2016. Available online: https://www.youtube.com/watch?v=EMD106bB2vY (accessed on 15 June 2021).

[36] Kobak D; Berens P The art of using t-SNE for single-cell transcriptomics. Nat. Commun 2019, 10, 1–14. 31780648 10.1038/s41467-019-13056-x PMC6882829

[37] Devassy B; George S Dimensionality reduction and visualisation of hyperspectral ink data using t-SNE. Forensic Sci. Int 2020, 311, 110194. 32251968 10.1016/j.forsciint.2020.110194

[38] Loukas S PCA Clearly Explained-When, Why, How to Use It and Feature Importance: A Guide in Python. 2016. Available online: https://towardsdatascience.com/pca-clearly-explained-how-when-why-to-use-it-and-feature-importance-a-guide-in-python-7c274582c37e#:~:text=In%20summary%2C%20PCA%20is%20an,each%20subsequent%20component%20explaining%20less (accessed on 15 June 2019).

[39] Ostwal P Principal Component Analysis Visualization. 2019. Available online: https://ostwalprasad.github.io/machine-learning/PCA-using-python.html (accessed on 15 June 2020).

[40] Wattenberg F; Viegas F; Johnson I How to Use t-SNE Effectively. Distill 2016. Available online: 10.23915/distill.00002 (accessed on 15 June 2021).

[41] Babcock CO; Bickel DL Constraint—The missing variable in the coal burst problem. In Proceedings of the 25th U.S. Symposium on Rock Mechanics, AIME, Northwestern University Chicago, Evanston, IL, USA, 25–27 June 1984; pp. 639–647.

